# Specific Distribution of the Autophagic Protein GABARAPL1/GEC1 in the Developing and Adult Mouse Brain and Identification of Neuronal Populations Expressing GABARAPL1/GEC1

**DOI:** 10.1371/journal.pone.0063133

**Published:** 2013-05-15

**Authors:** Jaclyn Nicole Le Grand, Karine Bon, Annick Fraichard, Jianhua Zhang, Michèle Jouvenot, Pierre-Yves Risold, Michaël Boyer-Guittaut, Régis Delage-Mourroux

**Affiliations:** 1 Université de Franche-Comté, Laboratoire de Biochimie, EA3922 «Estrogènes, Expression Génique et Pathologies du Système Nerveux Central», SFR IBCT FED 4234, U.F.R. Sciences et Techniques, Besançon, Doubs, France; 2 Department of Pathology, Center for Free Radical Biology, University of Alabama at Birmingham, Birmingham, Alabama, United States of America; 3 Department of Veterans Affairs, Birmingham VA Medical Center, Birmingham, Alabama, United States of America; IRB Barcelona, Parc Cientific de Barcelona and CIBERNED (ISCIII), University of Barcelona, Spain

## Abstract

Macroautophagy is a highly conserved cellular degradation process, regulated by autophagy-related (atg) factors, in which a double membrane autophagosome engulfs cytoplasmic components to target them for degradation. In yeast, the Atg8 protein is indispensable for autophagosome formation. In mammals, this is complicated by the presence of six Atg8 homologues grouped into the GABARAP and MAP1LC3 subfamilies. Although these proteins share a high similarity, their transcript expression, regulation and protein interactions differ, suggesting they may display individual properties and specific functions. GABARAPL1/GEC1 is a member of the GABARAP subfamily and its mRNA is the most highly expressed Atg8 homologue in the central nervous system. Consequently, we performed an in depth study of GABARAPL1 distribution in the developing and adult murine brain. Our results show that GABARAPL1 brain expression is visible as early as embryonic day 11 and progressively increases to a maximum level in the adult. Immunohistochemical staining was detected in both fibers and immature neurons in embryos but was restrained to neurons in adult tissue. By E17, intense punctate-like structures were visible and these accumulated in cortical primary neurons treated with the autophagosome/lysosome fusion inhibitor Bafilomycin A1 (Baf A1), suggesting that they represent autophagosomes. Finally, GABARAPL1 expression was particularly intense in motoneurons in the embryo and in neurons involved in somatomotor and neuroendocrine functions in the adult, particularly in the *substantia nigra pars compacta*, a region affected in Parkinson's disease. Our study of cerebral GABARAPL1 protein expression provides insight into its role in the development and homeostasis of the mouse brain.

## Introduction

Macroautophagy (hereafter referred to as autophagy), a degradation process conserved from plants and yeast to higher eukaryotes [1], has been described to play an essential role in cellular mechanisms such as lifespan extension, cellular development and differentiation [2] as well as in the development of several diseases including cancer, Huntington's, Alzheimer's and Parkinson's diseases [3], [4]. The yeast autophagy-related protein Atg8 is an ubiquitin-like modifier that localizes to and aids in the formation of autophagosomes [5], [6]. In mammals, the *atg8* family is divided into two subfamilies based on when they intervene in autophagosomal formation, the *MAP-lc3* subfamily (*Microtubule-Associated Protein light chain 3 A*, *B* and *C*) and the *gabarap* subfamily (*gabarap* for *GABA_A_ receptor-associated protein*; *gabarapl1/gec1* for *GABA_A_ receptor-associated protein like 1/glandular epithelial cells 1* and *gabarapl2/gate16* for *GABA_A_ receptor-associated protein like 2/GTPase activating protein of 16 kDa*) [7]. The LC3 subfamily is involved in earlier events in the process of autophagosomal formation and is necessary for elongation of the double membrane vesicle, whereas the GABARAP subfamily is implicated in latter stages of autophagosomal formation and is necessary for the closure of the vesicle [7]. The members of these two subfamilies, although similar, differ in their expression patterns, transcriptional regulation, and affinity for different protein partners, suggesting that their contribution to the autophagic pathway and even their particular role may vary depending on the tissue type and condition. To date, two *atg8* knockout mice have been created: for *lc3ß* [8] and *gabarap* [9]. Both knockout mice produce viable offspring and the *lc3ß* knockout mouse displays no difference in its autophagic phenotype, suggesting that in the absence of one family member, there is compensation by another. It is not known if the same is true for the members of the GABARAP subfamily since autophagy levels were not assessed in the *gabarap* knockout.

*gabarapl1/gec1*, one of the *gabarap* subfamily members, was originally discovered as an estrogen-regulated gene in guinea-pig glandular epithelial cells [10] and has since been identified as a member of the *gabarap* subfamily due to sequence similarity [11]. It is the most highly expressed transcript of the *atg8* mammalian homologues in the central nervous system [12], [13] and its mRNA expression has been mapped out in the rat central nervous system in 2008 [14]. *gabarapl1* mRNA expression varies throughout the rat brain, with a more abundant staining visible in neurons such as the pyramidal cells of the cerebral cortex and hippocampus, magnocellular neurons in the hypothalamus, purkinje cells of the cerebellum, and motoneurons in the brainstem and spinal cord, and a less expression visible in structures such as the striatum and the various reticular nuclei. In the same article, mRNA expression appeared to be limited to neurons, based on the lack of labelling in the fiber tracts and the morphology of the cells labelled in the gray matter.

At the protein level, GABARAPL1 expression has been identified by western blot analysis in different rat brain regions and confirmed in a few regions in which GABARAPL1 immunohistochemical labelling was visible such as the cortex, medial septal nucleus, diagonal band, hippocampus and motoneurons of the ventral horn [15]. In the latter study, authors used an antibody that appeared specific to GABARAPL1 in immunohistochemistry but that was not in immunoblotting techniques.

*gabarapl1* mRNA expression varies in different pathologies and following multiple stimuli. In the brain, a decrease in *gabarapl1* transcript levels have been observed in both the cortex of macaques treated with MPTP (1-methyl-4-phenyl-1,2,3,6- tetrahydropyridine), a neurotoxin which mimics the effect of the development of Parkinson's disease in animal models, and in the *substantia nigra* neurons of human Parkinson's patients [16], [17].

We recently set out to study the specificity of several antibodies directed against GABARAPL1 and identified an antibody that is specific to the latter protein in both western blot and immunohistochemical experiments [18]. With this tool in hand, we have mapped out the protein expression of GABARAPL1 in the adult mouse brain and during the development. Our results demonstrate a protein expression throughout the adult mouse brain similar to the *gabarapl1* mRNA distribution previously described in rat. During development and in the adult mouse, GABARAPL1 is distributed throughout the brain, from the olfactory bulb to brainstem, with varying intensity. Although GABARAPL1 is found only in neurons in the mature brain, it is also observed in fiber tracts during neuronal development. At a cellular level, GABARAPL1 displays a weak cytoplasmic and sometimes dendritic staining with stronger intensity punctate structures clustered within the cytoplasm. These structures are increased in the presence of a drug that inhibits the autophagy flux and the majority co-localizes with the specific autophagic substrate p62, suggesting that they are indeed autophagosomes. GABARAPL1 staining was observed in long projection neurons and interneurons, and co-localized with antibodies against choline acetyl transferase (ChAT), the dopaminergic transporter (DAT), calbindin and parvalbumin. This study describes for the first time the expression of an autophagic protein, GABARAPL1, in the mouse brain and during its development. Our data demonstrate that this expression is not ubiquitous and shows high differences throughout the mouse brain. These data suggest that the basal autophagy levels might vary in the different types of neurons at different stages of their life and that this process might be highly regulated.

## Materials and Methods

### Animals

Cerebral tissues from wild type (WT) embryo, WT adult male mice and GABARAP−/− adult male mice were used for GABARAPL1 protein expression mapping. WT Swiss mice were obtained from Janvier, Le Genest-Saint-Isle, France. GABARAP−/− 129/SvEv-C57BL/6 mice were kindly provided by Pr. Heinrich Betz, Department of Neurochemistry, Max-Planck-Institute for Brain Research, Frankfurt, Germany. The time of conception for embryos was documented by vaginal plug observation (embryonic day 0, E0). Timed pregnant mice and adult male mice were euthanized by cervical dislocation. Mice pups at E11, E12, E13, E14 and E17 were sacrificed by decapitation.

### Tissue fixation and cryoprotection

Adult male mice were perfused as previously described [19] with 0.9% NaCl followed by ice- cold 1% paraformaldehyde (PFA, ROTH) fixative in PBS (0.137 M NaCl, 3.3 mM KCl, 10 mM Na_2_HPO4, 1.8 mM KH_2_PO4). For immunohistochemistry experiments, extracted adult and embryonic brains were post-fixed in 1% PFA for several hours at 4°C and cryoprotected by saturation in a 15% sucrose solution (Sigma, 17 994-9) at 4°C overnight. Fresh tissue contained within an Eppendorf tube (for western-blotting experiments) or cryoprotected tissue (for immunohistochemistry experiments) was frozen by immersion in isopentane using the Snap-Frost**™** system (Excilone, France), sectioned at −20°C into 10 µm sections using a cryostat-microtome (Microm), placed on gelatin-coated slides (ROTH) and stored at −40°C.

### Immunohistochemistry

For immunohistochemical staining, slides were prepared by rehydration in PBS-T (PBS with 0.3% Triton X100) for three 5 min washes at room temperature. Tissue was subsequently incubated with the appropriate primary antibody dissolved in milk diluant (PBS-T containing 1% bovine serum albumine, 10% lactoproteins and 0.01% sodium azide) overnight at room temperature. The primary anti-GABARAPL1 specific antibody used was anti-ATG8L (rabbit polyclonal, Proteintech, 11010-1-AP, 1∶500) [18]. Slides were then washed three times with PBS-T for 5 min each at room temperature before being incubated with the appropriate secondary antibody diluted in milk solution for 1 h at room temperature. The secondary antibody used was the Alexa Fluor 488 goat anti-rabbit IgG (Invitrogen, 1∶800). Finally, the slides were washed with PBS-T three times 5 min and cover slips were mounted with 70∶30 glycerol∶PBS-T.

### Immunostaining of mouse embryonic cortical primary neurons

Dissected cortices from 8 embryos were resuspended in 5 ml of HBSS (Invitrogen) and then incubated with 26 U/ml of Papain (Worthington) for 20 min at 37°C. After addition of 2 ml of NBM+ medium (Neurobasal medium, X B27, 100 µg/ml Penicillin-Streptomycin, 0.5 mM L-Glutamine, Invitrogen), the cortices were grounded and centrifuged for 5 minutes at 1000 g. The pellet was then resuspended in 2 ml of complete NBM+ medium and 500 µl were loaded on 2 ml of a 4% BSA solution before a centrifugation step at 1000 g for 5 min to remove debris. The pellet was then resuspended in 2 ml NBM+ medium and the cell concentration determined before plating on coverslips in 24 well plates at a concentration of 2.10^5^ cells per well. The primary neurons were then cultured in an incubator at 37°C in a 5% CO_2_ atmosphere for 7 days with half the medium changed on day 2 and 5. On day 7, half of the medium was replaced with fresh medium with or without Baf A1 (Sigma) at a final concentration of 100 nM and incubated for a further 16 h. The cells were then washed once in PBS and fixed for 45 min in a 4% PFA solution in PBS. Cells were then washed twice in PBS and subsequently permeabilized for 3 min in a 0.5% Triton-X-100 solution in PBS, washed four times with PBS, blocked for 1 h in 3% BSA in PBS and then incubated overnight at 4°C with the anti- ATG8L (rabbit polyclonal, Proteintech, 11010-1-AP, 1∶500), anti-p62 (mouse monoclonal, Abnova, 1∶500), anti-GFAP (rabbit polyclonal, Dako, 1∶500) and the anti-NeuN antibody (mouse monoclonal, Millipore, 1∶500) dissolved in blocking buffer. Coverslips were then washed three times with PBS for 5 min each at room temperature before being incubated with an Alexa Fluor 488 goat anti-rabbit IgG (Invitrogen, 1∶500) and an Alexa Fluor 568 goat anti-mouse IgG (Invitrogen, 1∶500) diluted in PBS for 1 h at room temperature in the dark. Finally, the cells were washed four times 5 min with PBS, incubated with a 2 µg/ml Hoescht solution in PBS for 10 min at room temperature, washed once in PBS and coverslips were mounted on slides in Fluoromount-G (Southern Biotechnology Associates, Inc).

### Microscopy

Fluorescent tissue sections were examined with either a fluorescent microscope (BX51, Olympus, France), a fluorescence laser scanning confocal microscope (IX81, Olympus, France, SFR IBCT FED 4234 microscopy core Besançon, France) or a Zeiss LSM 710 confocal microscope (UAB microscopy core, Birmingham, AL, USA). Images were captured with either a DP50 or DP75 numeric cameras (Olympus, France) using the Fluoview FV1000 software, AnalySIS3.1 software (Soft Imaging System) or Zen 2008 software, respectively. All figures were assembled using Adobe Photoshop CS2 and Adobe Illustrator CS software.

### Western blot analysis

Brain tissue was ground and lysed using the following lysis buffer: [10 mM Tris-HCl pH 7.4, 100 mM NaCl, 5 mM EDTA, 10 mM MgCl_2_, 0.5% NP-40, 1% Triton X-100, protease inhibitor cocktail (Sigma, P8340)] then 40 µg of tissue lysates were resolved on a 15% polyacrylamide gel in running buffer (25 mM Tris base, 200 mM glycine and 0.1% SDS) at 20 mA using a Biorad Power Pack 1000. Proteins were transferred onto Immun-Blot PVDF 0.2 µm membranes (Biorad) for 1 h at 4°C at 200 mA in Western Blot transfer buffer (25 mM Tris base, 200 mM glycine and 10% methanol). Membranes were subsequently blocked in TBS-T (199 mM Tris-HCl, pH 7.4, 1.36 mM NaCl, 0.1% Tween 20) with 5% skim milk powder. Membranes were blotted in TBS-T supplemented with 0.5% skim milk and anti-ATG8L (rabbit polyclonal, Proteintech, 1∶1000) and anti-Actin (rabbit polyclonal, Sigma, 1∶3000). The secondary antibody horseradish peroxydase-coupled anti-rabbit IgG was prepared in TBS-T containing 0.5% skim milk powder. Binding of antibodies to membranes was detected by Enhanced Chemiluminescence Plus Reagent (ECL Plus, GE Healthcare Life Sciences), according to the manufacturer's protocol. . Image Lab software (Bio-Rad Laboratories) was used to quantify protein band density.

### Statistical analysis

Statistical analyses were carried out using a Student's t test on GraphPad PRISM® software. Data are expressed as the mean ± SEM, **p<0.05 versus E11, *p<0.1 versus E11 (n = 3).

### Ethics Statement

The appropriate institutional ethics committee approved all experiments in this study. All animal care and experimental protocols adhere to the institutional guidelines and are in compliance with the University of Alabama at Birmingham and the University of Franche-Comte Institutional Animal Care and Use Committee guidelines.

## Results

### Anti-GABARAPL1 antibody specificity and staining

Since GABARAPL1 shares a very high sequence identity (86% in the case of the human proteins) with its closest homologue GABARAP, we used the only specific anti-GABARAPL1 antibody, to our knowledge, available on the market for our study. This antibody from Proteintech is specific of GABARAPL1, not only in western blot but also in immunohistochemistry experiments [18].

**Figure 1:**
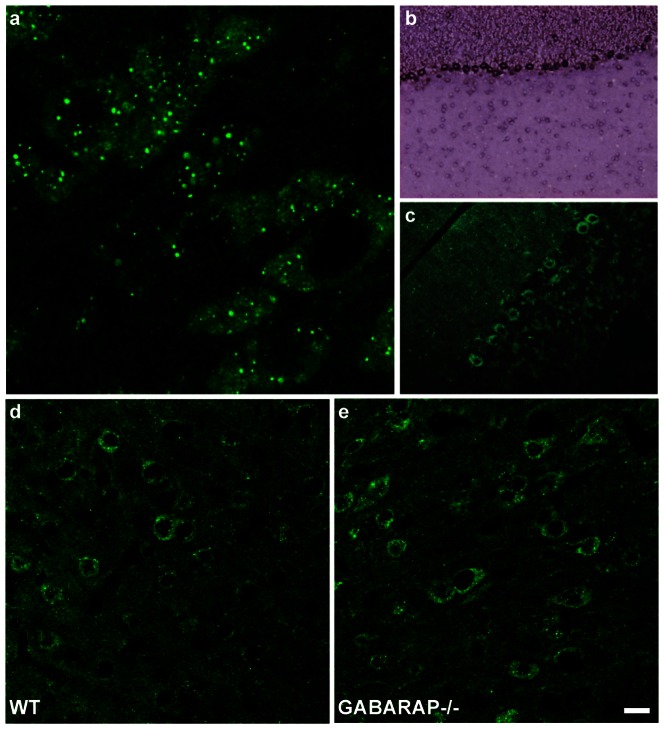
GABARAPL1 protein and *gabarapl1* mRNA staining in WT and GABARAP−/− mice. Immunohistochemical and *in-situ* hybridization analysis of WT and GABARAP−/− cortical mouse brain tissue with an anti-GABARAPL1 antibody (**a**, **c–e**) and an anti-sense *gabarapl1* Dig-labelled probe (**b**). Anti-GABARAPL1 antibody displays a diffused cytoplasmic staining with brighter punctate structures (**a**: neurons of the DMX). *gabarapl1* mRNA and GABARAPL1 protein expression patterns were similar throughout the brain (**b**, **c**: Purkinje cells of the cerebellum). Anti-GABARAPL1 staining patterns remained the same between wild type and GABARAP knock out mice brains (**d**, **e**: cerebellar cortex). Scale bar represents 5 µm (**a**), 80 µm (**b**), 40 µm and 20 µm (**d**, **e**).

At a cellular level, GABARAPL1 localization is displayed as weak labelling throughout the cytoplasm and some neuronal extensions with brighter punctate labelling within the cell body (Fig. 1a in adult and 12d in embryo). This pattern is observed in all the labelled neurons throughout the adult brain, but is more prominent at E17 than E11 in the embryonic brain. This localization is similar to the one observed previously in cellular overexpression models [20], [21] and is consistent with its previously described roles in receptor transport and the formation of autophagosomes.

Although our study was performed using the same antibody lot previously demonstrated to be specific to GABARAPL1, we nevertheless took supplementary precautions to confirm the specificity of the staining observed. Firstly, we performed *in-situ* hybridization on the preceding slide in the wild type adult or embryonic mouse series with a probe recognizing a 3′-UTR region of *gabarapl1* mRNA whose specificity has been previously confirmed [14]. These experiments allowed us to correlate the mRNA *gabarapl1* expression to its protein expression in the same region (Fig. 1b–c). Secondly, we studied the expression of GABARAPL1 in the GABARAP knockout mouse to confirm its distribution pattern. To do so, we performed immunohistochemical experiments, using the same GABARAPL1 antibody, on a series of coronal sections produced from a GABARAP knockout mouse or GABARAP knockout embryos and verified the features of the staining produced in the different regions examined in the wild type mouse brain. In all the regions examined, the GABARAPL1 staining pattern was found to be the same as the one observed in the wild type animals (Fig. 1d–e).

### GABARAPL1 expression in embryonic cortical primary cultures

**Figure 2:**
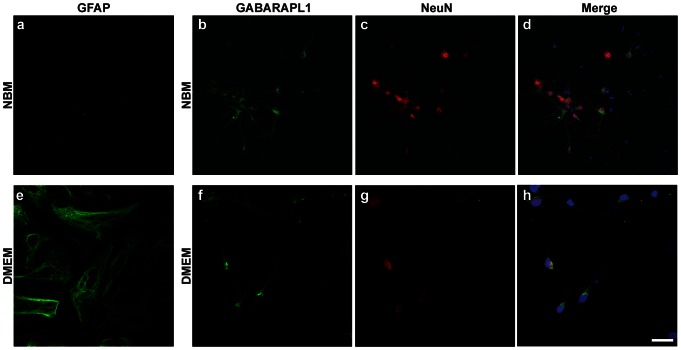
GABARAPL1 expression in embryonic cortical primary cultures grown in NBM or DMEM medium. Immunostaining using anti-GFAP (green), anti-GABARAPL1 (green) or anti-NeuN (red) of embryonic cortical primary neurons grown in NBM or Dulbecco's Modified Eagle Medium (DMEM) supplemented with 10% FBS. GABARAPL1 staining is abundant under conditions that support neuronal cell differentiation (NBM grown, GFAP-negative cells) and scarce under conditions that support glial cell differentiation (DMEM grown, GFAP-positive cells). All GABARAPL1 positive cells are also labeled with NeuN. Scale bar represents 40 µm (**a**, **e–f**) and 80 µm (**b–d**).

The GABARAPL1 labeling pattern in the whole adult brain revealed that this protein, like its mRNA, is found predominantly in the grey matter and thus likely only within neurons. In order to confirm the neuronal expression of GABARAPL1, we utilized mouse embryonic cortical primary cell cultures to study GABARAPL1 staining in an *ex vivo* model. We performed immunocytofluorescence experiments with our specific anti-GABARAPL1 antibody and a marker of neuronal cell types, anti-NeuN (neuronal nuclei), or a marker of glial cells, anti-GFAP (glial fibrillary acidic protein), on primary cell cultures grown in either neurobasal media (NBM, Fig. 2a–d) or Dulbecco's Modified Eagle Medium supplemented with 10% FBS (DMEM, Fig. 2e–h), producing primarily neuronal or glial cell cultures, respectively. In both *ex vivo* models, all GABARAPL1 positive cells were also positive for NeuN. Consistent with the small percentage of neuronal cells in these cultures, very little GABARAPL1 staining was visible in the DMEM grown cultures (predominantly glial cells). Together, these results demonstrate that GABARAPL1 protein expression is found primarily in neurons.

**Figure 3:**
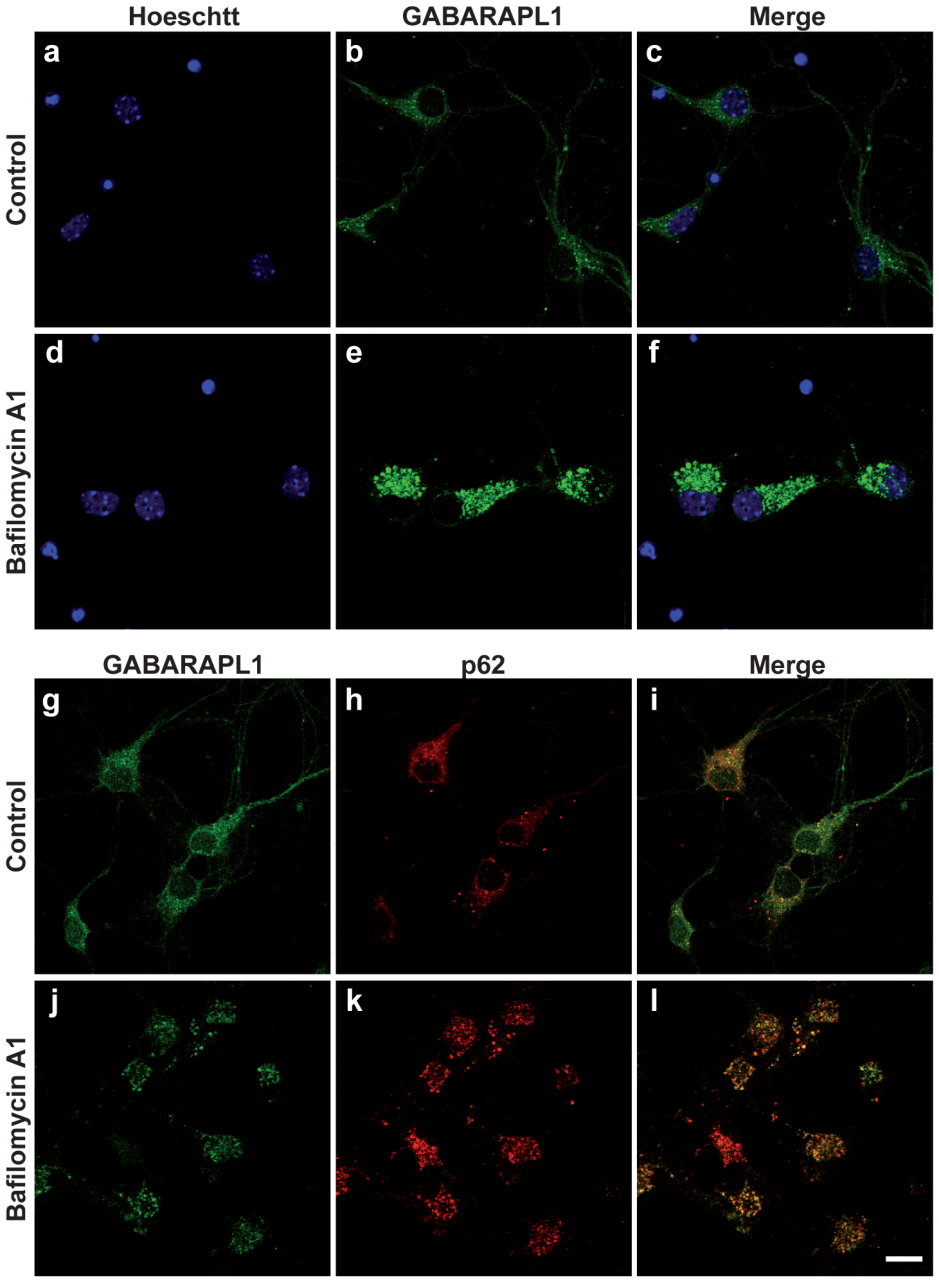
GABARAPL1 expression in embryonic cortical primary cultures after Baf A1 treatment. Immunostaining using anti-GABARAPL1 (green) and anti-p62 (red) of embryonic cortical primary neurons incubated with or without Baf A1, a described inhibitor of the autophagic flux. The control cells show a diffuse and punctiform staining of GABARAPL1 and p62 in the cell body as well as in the cell processes. The incubation of the cells with Baf A1 induces an important increase in the number of GABARAPL1 and p62-positively stained vesicles suggesting that the GABARAPL1 staining observed in neurons is due to its association with autophagic vesicles. Scale bar represents 20 µm.

Since GABARAPL1 has been previously described to be involved in receptor transport in neurons [21], [22] and to be associated with autophagic vesicles [20], the punctate staining observed in the neurons of the mouse brain is most likely associated with secretion or autophagic vesicles. Previously published studies have demonstrated that the visualisation of autophagic proteins such as p62 or LC3 in the normal brain is difficult. Indeed, these studies describe a lack of p62 or punctate LC3 staining in normal brain tissue [23], [24], [25]. Like these other studies, we were unable to identify p62 labeling or LC3 punctate labeling in the mouse brain tissue. As such, we performed co-labeling experiments in primary neuronal cultures. To determine whether GABARAPL1 is linked to autophagic vesicles in neurons, we incubated the primary cultures in the presence of Baf A1, a described inhibitor of the autophagic flux, allowing the accumulation of autophagic vesicles in the cells [26]. Like the *in vivo* staining described earlier, GABARAPL1 displayed a weak cytoplasmic and a stronger punctiform staining in primary neuron cultures (Fig. 3b). When the autophagic flux is blocked with Baf A1, we observed an increase in the number of GABARAPL1-positive vesicles in the cells (Fig. 3e), suggesting that the punctiform staining observed in these neurons, and therefore in the mouse brain, is most likely linked to autophagic vesicles. We then stained these primary neuron cultures with an anti-p62 antibody. p62 has been described as a protein specifically degraded through the autophagosome/lysosome pathway and shown to accumulate in autophagosomes in presence of BafA1 [27]. As seen in Fig. 3g–i, in control cells, GABARAPL1 and p62 staining are diffuse throughout the cytoplasm with some more intense vesicle staining and we observe little co-localization. Conversely, when the cells are incubated with BafA1 (Fig. 3j–l), we observe an increase in the number of these vesicles and an accumulation of the two proteins in most, but not all, of these structures. We can therefore conclude that the GABARAPL1 staining observed in mouse neurons is primarily linked to autophagosomal structures.

### Distribution of GABARAPL1 protein in the adult brain

**Figure 4:**
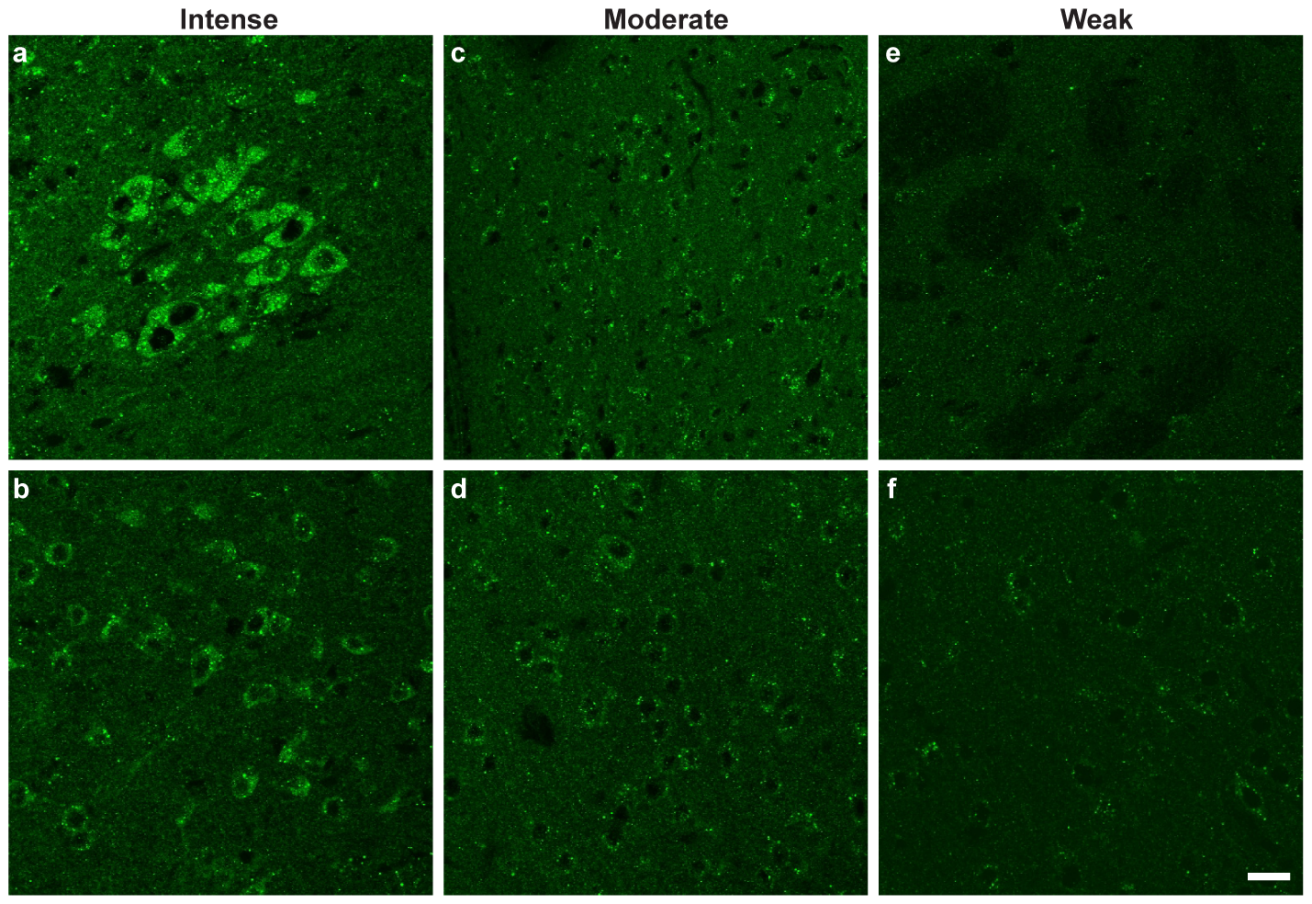
Cellular localization of GABARAPL1 in various cerebral regions. Immunohistochemical analysis of WT mouse brain tissues with an anti-GABARAPL1 antibody (**a–f**). Representative images of intense labelling in the dorsal motor nucleus of the vagus nerve- DMX (**a**) and nucleus of the diagonal band- NDB (**b**), moderate labelling in the paraventricular nucleus of the hypothalamus PVH (**c**) and the cerebellar cortex (**d**) and weakly labelled neurons in the caudate putamen (**e**) and the globus pallidus (**f**). Scale bar represents 20 µm.

The protein expression of GABARAPL1 in the brain mapped out in this study describes an expression pattern congruent with that of its mRNA [14]. We confirmed that the GABARAPL1 protein is abundantly expressed in the brain from the olfactory bulb to the brainstem. GABARAPL1 distribution, however, was not uniform throughout the brain and the intensity of labelling varied in labelled cells. A detailed list of the specific brain regions labelled with the anti-GABARAPL1 antibody is displayed in Table 1. Cells found to be positive for GABARAPL1 were designated by a relative level of staining from 0 to +++ based on their intensity. Examples of intense (Fig. 4a–b), moderate (Fig. 4c–d) and weakly (Fig. 4e–f) labelled cells can be seen in Figure 3.

**Table 1:** Distribution of GABARAPL1 in the Mouse Brain.

Region	Expression
**CEREBRUM**	
**CEREBRAL CORTEX**	
isocortex, layer 1–6 (ISO1–6)	++/+++
main olfactory bulb (MOB)	+/++
accessory olfactory bulb (AOB)	+/++
anterior olfactory nucleus (AON)	+/++
taenia tecta (TT)	++
piriform area (PIR)	0/++
nucleus of the lateral olfactory tract, pyramidal layer (NLOT2)	++
cortical nucleus of the amygdala (COA)	++
agranular insular area (AI)	++/+++
anterior cingulate area (ACA)	++/+++
ectorhinal area (ECT)	++/+++
Retrohippocampal region (RHP)	
entorhinal area (ENT)	++/+++
presubiculum (PRE)	++/+++
postsubiculum (POST)	++/+++
parasubiculum (PAR)	++/+++
Subiculum, pyramidal layer (SUB-sp)	++/+++
Hippocampal region (HIP)	
Ammon's horn: field CA1–3 (CA1–3)	
stratum lacunosum-moleculare (-slm)	0
stratum radiatum [Meynert] (-sr)	0
pyramidal layers (-sp)	++/+++
stratum oriens (-so)	0
stratum lucidum (CA3slu)	0
Dentate gyrus: crest (DGcr), lateral blade (DGlb), medial blade (DGmb)	
molecular layers (-mo)	0
granule cell layers (-sg)	++/+++
polymorph layers (-po)	+
induseum griseum (IG)	++
fasciola cinerea (FC)	+
Infralimbic area (ILA)	++/+++
Orbital area (ORB)	++/+++
Perihinal area (PERl)	++/+++
Prelimbic area (PL)	++/+++
Retrosplenial area (RSP)	++/+++
Ventral temporal association areas (TEv)	++/+++
Layer 6b, isocortex (6b)	+++
Claustrum (CLA)	+++
Endopiriform nucleus (EP)	++/+++
Basolateral nucleus of the amygdala (BLA)	+
Basomedial nucleus of the amygdala (BMA)	0/+
Posterior nucleus of the amygdala (PA)	0/+
**BASAL NUCLEI**	
caudoputamen (CP)	+
nucleus accumbens (ACB)	+
fundus of the striatum (FS)	0/+
olfactory tubercle (OT)	++/+++
islands of Calleja (isl)	0/+
major island of Calleja (islm)	0/+
lateral septal nucleus (LS)	++
septofimbrial nucleus (SF)	+
septohippocampal nucleus (SH)	0/+
anterior amygdaloid area (AAA)	+
central nucleus of the amygdala (CEA)	+
medial nucleus of the amygdala (MEA)	++
bed nucleus of the accessory olfactory tract (BA)	+
intercalated nucleus of the amygdala (IA)	+
globus pallidus (GP)	0/+
substantia innominata (SI)	+/++
magnocellular preoptic nucleus (MA)	+++
medial septal nucleus (MS)	+++
nucleus of the diagonal band (NDB)	+++
triangular nucleus of the septum (TRS)	+
bed nucleus of the stria terminalis (BST)	+/++
bed nucleus of the anterior commissure (BAC)	0/+
bed nucleus of the stria medullaris (BSM)	0/+
**CEREBELLUM**	
**CEREBELLAR CORTEX (CBX)**	
Vermal regions (VERM)	+/++
Hemispheric regions (HEM)	+/++
**DEEP CEREBELLAR NUCLEI (DNC)**	
Fastigial nucleus (FN)	+++
Interposed nucleus (IP)	++/+++
Dentate nucleus (DN)	++
**BRAINSTEM**	
**THALAMUS (TH)**	
anteroventral nucleus of the thalamus (AV)	++
anteromedial nucleus of the thalamus (AM)	+
anterodorsal nucleus of the thalamus (AD)	+++
mediodorsal nucleus of the thalamus (MD)	+
paraventricular nucleus of the thalamus (PVT)	++
nucleus reuniens (RE)	+
central medial nucleus of the thalamus (CM)	+
paracentral nucleus of the thalamus (PCN)	+
parafascicular nucleus (PF)	+
suprageniculate nucleus (SGN)	+
ventral anterior-lateral complex of the thalamus (VAL)	+
ventral posterolateral nucleus of the thalamus (VPL)	+
ventral posteromedial nucleus of the thalamus (VPM)	+
Medial geniculate complex (MG)	+/++
Medial habenula (MH)	+/++
Lateral habenula (LH)	+
Reticular nucleus of the thalamus (RT)	++
Zona incerta (ZI)	+
**HYPOTHALAMUS**	
**Periventricular zone of the hypothalamus (PVZ)**	
vascular organ of the lamina terminalis (OV)	+
suprachiasmatic preoptic nucleus (PSCH)	+/++
median preoptic nucleus (MEPO)	+/++
anteroventral periventricular nucleus (AVPV)	++/+++
preoptic periventricular nuclueis (PVpo)	+
supraoptic nucleus (SO)	++
paraventricular nucleus of the hypothalamus (PVH)	++/+++
anterior periventricular nucleus of the hypothalamus (PVa)	+
dorsomedial nucleus of the hypothalamus (DMH)	+/++
intermediate periventricular nucleus of the hypothalamus (PVi)	+
arcuate nucleus of the hypothalamus (ARH)	+++
posterior periventricular nucleus of the hypothalamus (PVp)	+
**Medial zone of the hypothalamus (MEZ)**	
medial preoptic area (MPO)	+/++
medial preoptic nucleus (MPN)	++/+++
anterodorsal preoptic nucleus (ADP)	+/++
anteroventral preoptic nucleus (AVP)	++
suprachiasmatic nucleus (SCH)	+
subparaventricular zone (SBPV)	+
ventromedial nucleus of the hypothalamus (VMH)	++
premammillary nucleus (PM)	++
tuberomammillary nucleus (TM)	+++
supramammillary nucleus (SUM)	+/++
medial mammillary nucleus (MM)	++/+++
lateral mammillary nucleus (LM)	+++
posterior hypothalamus nucleus (PH)	+
**Lateral zone of the hypothalamus (LZ)**	
lateral preoptic area (LPO)	+/++
lateral hypothalamus area (LHA)	++/+++
tuberal nucleus (TU)	+
subthalamic nucleus (STN)	++
**MIDBRAIN-HINDBRAIN**	
**SENSORY**	
superior colluculus (SC)	0/++
parabigemical nucleus (PBG)	+/++
olivary pretectal nucleus (OP)	+
nucleus of the optic tract (NOT)	+
posterior pretectal nucleus (PPT)	+
nucleus of the posterior commissure (NPC)	0/+
anterior pretectal nucleus (APN)	+
medial pretectal area (MPT)	+
medial terminal nucleus of the accessory optic tract (MT)	+
lateral terminal nucleus of the accessory optic tract (LT)	+
dorsal terminal nucleus of the accessory optic tract (DT)	+
trigeminal ganglion (GV)	++/+++
mesencephalic nucleus of the trigeminal (MEV)	++/+++
principle sensory nucleus of the trigeminal (PSV)	+
spinal nucleus of the trigeminal (SPV)	+
paratrigeminal nucleus (PAT)	+/++
dorsal column nuclei (DCN)	+
external cuneate nucleus (ECU)	+++
cochlear nuclei (CN)	+
dorsal nucleus (DCO)	+
ventral nucleus (VCO)	+++
nucleus of the trapezoid body (NTB)	+++
superior olivary complex (SOC)	+++
nucleus of the lateral lemniscus (NLL)	++
interior colluclus (IC)	+
nucleus of the brachium of the inferior colliculus (NB)	0/+
nucleus sagulum (SAG)	+/++
medial vestibular nucleus (MV)	+
lateral vestibular nucleus (LAV)	++
superior vestibular nucleus (SUV)	0/+
spinal vestibular nucleus (SPIV)	++
nucleus intercalatus (NIS)	++/+++
nucleus prepositus (PRP)	0/+
nucleus of Roller (NR)	+/++
nucleus x (x)	++
nucleus (y)	++
infracerebellar nucleus (ICB)	++
nucleus of the solitary tract (NTS)	+
area postrema (AP)	0/+
parabrachial nucleus (PB)	++
Kölliker-Fuse subnucleus (KF)	++
**MOTOR**	
oculomotor nucleus (III)	++
trochlear nucleus (IV)	++
abducens nucleus (VI)	++
accessory abducens nucleus (ACVI)	++
motor nucleus of the trigeminal (V)	++
parvicellular part (Vpc)	++
facial nucleus (VII)	++
accessory facial nucleus (ACVII)	++
efferent vestibular nucleus (EV)	++/+++
nucleus ambiguous, dorsal division (AMBd)	++
hypoglossal nucleus (XII)	+++
Edinger-Westphal nucleus (EW)	0/+
superior salivatory nucleus (SSN)	+
inferior salivatory nucelsu (ISN)	+
dorsal motornucleus of the vagus nerve (DMX)	+++
nucleus ambiguous, ventral division (AMBv)	++
compact part (SNc)	+++
reticular part (SNr)	+
ventral tegmental area (VTA)	+++
**PRE- & POSTCEREBELLAR NUCLEI**	
Pontine gray (PG)	++/+++
tegmental reticular nucleus (TRN)	+
Inferior olivary complex (IO)	++/+++
Lateral reticular nucleus (LRN)	++/+++
Linear nucleus of the medulla (LIN)	++
Paramedian reticular nucleus (PMR)	++
Parasolitary nucleus (PAS)	+
Red nucleus (RN)	++/+++
**RETICULAR CORE**	
periaqueductal gray (PAG)	0/+
interstitial nucleus of Cajal (INC)	+
nucleus of Darkschewitsch (ND)	+
dorsal tegmental nucleus (DTN)	++
ventral tegmental nucleus (VTN)	++
anterior tegmental nucleus (AT)	+/++
lateral tegmental nucleus (LTN)	++
laterodorsal tegmental nucleus (LDT)	++
sublaterodorsal nucleus (SLD)	++
locus coeruleus (LC)	++
subcoeruleus nucleus (SLC)	++
Barrington's nucleus (B)	++
supragenual nucleus (SG)	++/+++
pontine central gray (PCG)	++
Raphé nuclei (RA)	+/++
nucleus incertus (NI)	++
Reticular formation (RET)	+/++

GABARAPL1 protein expression in different regions of the mouse CNS is displayed with a relative quantification of the intensity of staining observed. 0: absence of labeling, +: weak intensity, ++ moderate intensity, +++: strong intensity.

#### Telencephalon

**Figure 5:**
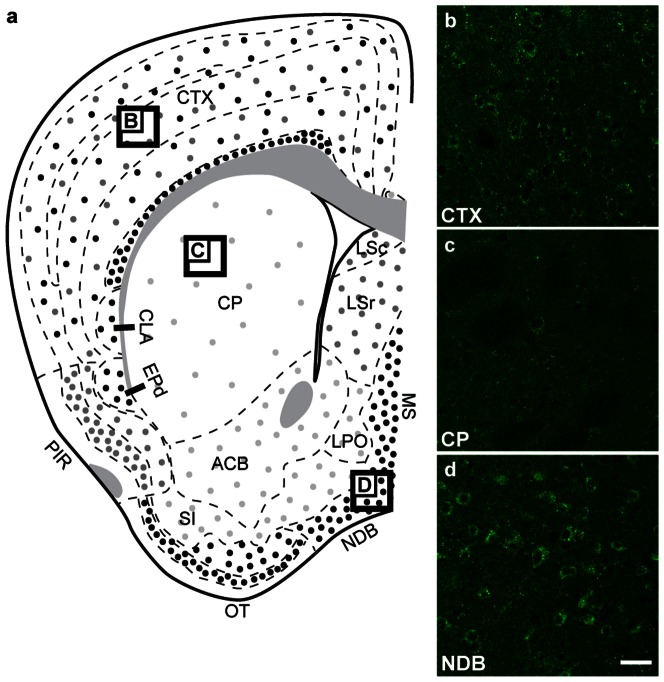
GABARAPL1 expression in the telencephalon. Immunohistochemical analysis of WT mouse brain tissue with an anti-GABARAPL1 antibody. Images represent typical GABARAPL1 protein expression patterns in neurons found in the cortex-CTX (**b**), caudate putament-CP (**c**) and nucleus of the diagonal band-NDB (**d**). Scale bar represents 20 µm. Abbreviations: ACB (nucleus acumbens), CLA (claustrum), CP (caudate putamen), CTX (cortex), EPd (dorsal endopiriform nucleus), IG (induseumgriseum), LPO (lateral preoptic area), LSr (lateral septal nucleus- rostral part), LSc (lateral septal nucleus- caudal part), MS (medialis strialis), NDB (nucleus of the diagonal band), OT (olfactory tract), PIR (piriform area), SI (substantia innominata).

GABARAPL1 expression was observed throughout the telencephalon, like its mRNA, with a higher expression found in the cerebral cortex and moderate to weak expression observed in most of the basal nuclei with the exception of the magnocellular preoptic and medial septal nuclei and the nucleus of the diagonal band, in which very intensely labelled cells were observed. GABARAPL1 protein distribution within the telencephalon is depicted in Fig. 5a with representative images of staining in the cerebellar cortex (Fig. 5b), the striatum (Fig. 5c), and nucleus of the diagonal band (Fig. 5d).

**Figure 6:**
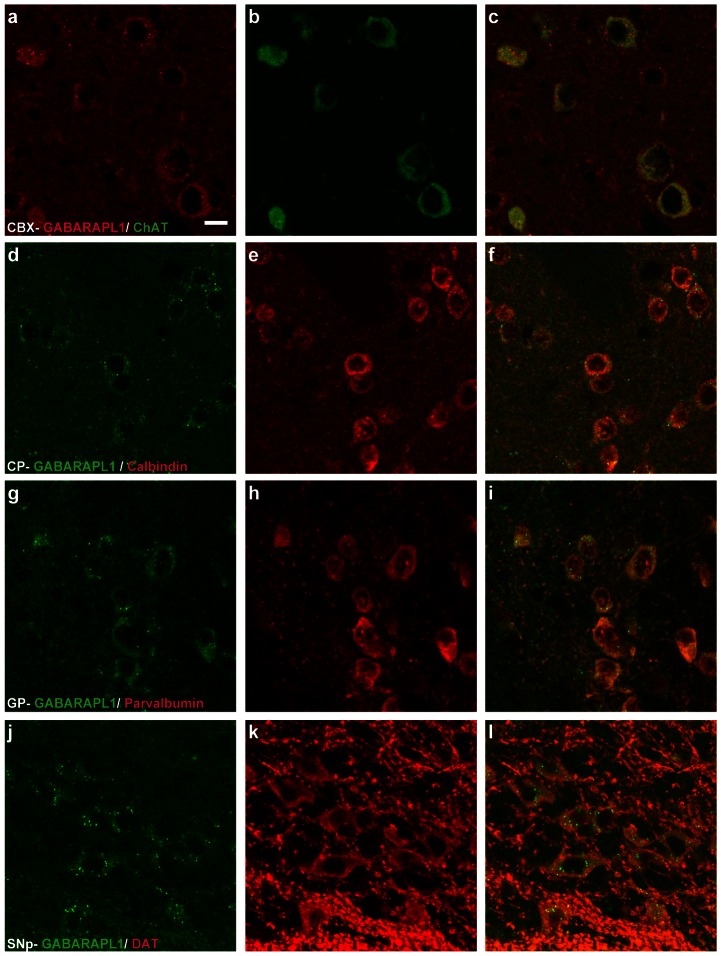
GABARAPL1 expression in different neuronal populations. Immunohistochemical analysis of WT mouse brain tissue with an anti-GABARAPL1 antibody and different neuronal population markers. Images represent double staining with anti-GABARAPL1 and either anti-choline acetyltransferase in the cerebellar cortex-CBX (**a–c**), anti-calbindin in the caudate putamen-CP (**d–f**), anti-parvalbumin in the globus pallidus-GP (**g–i**) or anti-dopamine transporter in the *substantia nigra*, *pars compacta*-SNpc (**j–l**). Scale bar represents 10 µm.

Within the telencephalon, GABARAPL1 expression was identified in cholinergic neurons visible in the cerebral cortex (ChAT-positive cells, Fig. 6a–c) the striatum and the pallidum as well as calbindin and parvalbumin-positive cells in the striatum (Fig. 6d–i).

#### Diencephalon

**Figure 7:**
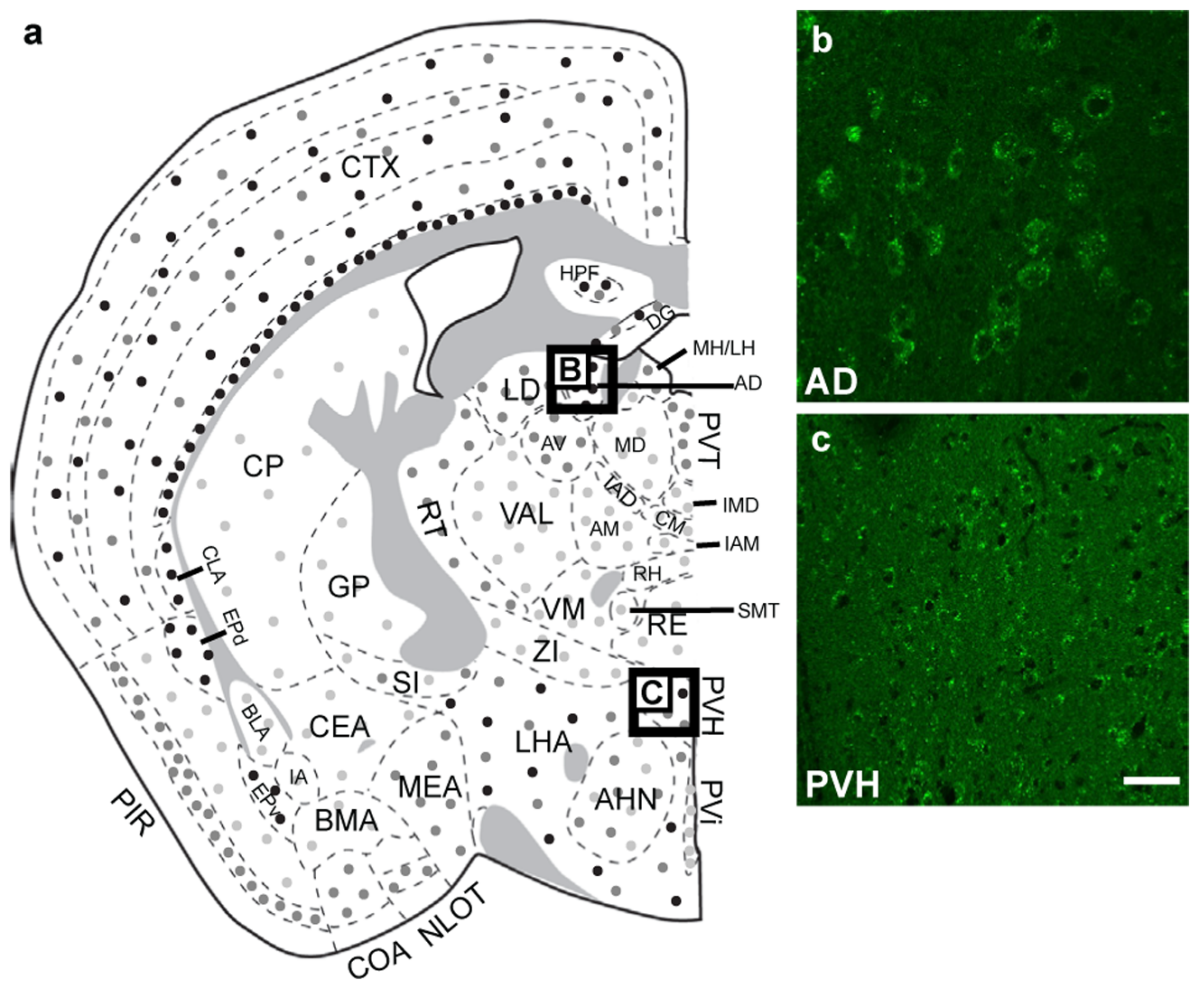
GABARAPL1 expression within the diencephalon. Immunohistochemical analysis of WT mouse brain tissue with an anti-GABARAPL1 antibody. Images represent typical GABARAPL1 protein expression patterns in neurons found in the anterodorsal nucleus of the thalamus-AD (**b**) and paraventricular nucleus of the hypothalamus-PVH (**c**). Scale bar represents 20 µm. Abbreviations: AD (anterodorsal nucleus of the thalamus), AHN (anterior hypothalamic nucleus), AM (anteromedial nucleus of the thalamus), AV (anteroventral nucleus of the thalamus), BLA (basolateral nucleus of the amygdala), BMA (basomedial nucleus of the amygdala), CEA (central nucleus of the amygdala), CLA (claustrum), CM (central medial nucleus of the thalamus), COA (cortical nucleus of the amygdala), CP (caudate putamen), CTX (cortex), DG (dentate gyrus), EPd (endopiriform nucleus- dorsal part), EPv (endopiriform nucleus-ventral part), GP (globus pallidus), HPF (hippocampal formation), IA (intercalated nucleus of the amygdala), IAD (interanterodorsal nucleus of the thalamus), IAM (interanteromedial nucleus of the thalamus), IMD (intermediodorsal thalamic nucleus), LD (lateral dorsal nucleus of the thalamus), LH (lateral habenula), LHA (lateral hypothalamus area), MD (mediodorsal nucleus of the thalamus), MEA (medial nucleus of the amygdala), MH (medial habenula), NLOT (nucleus of the lateral olfactory tract), PIR (piriform area), PVH (paraventricular nucleus of the hypothalamus), PVT (paraventricular nucleus of the thalamus), RE (nucleus reuniens), RH (rhomboid nucleus), RT (reticular nucleus of the thalamus), SI (substantia innominata), SMT (submedial nucleus of the thalamus), VAL (ventral anterior-lateral complex of the thalamus), VM (ventral medial nucleus of the thalamus), ZI (zona incerta).

GABARAPL1 protein distribution within the diencephalon is depicted in Fig. 7a. GABARAPL1 expression could be seen throughout the diencephalon with varying intensities. Except for the anterodorsal nucleus of the thalamus (AD), (Fig. 7b), the thalamus was only weakly to moderately stained with anti-GABARAPL1. GABARAPL1 expression was overall higher in the hypothalamus, particularly in the paraventricular nucleus (PVH) (Fig. 7c), the medial preoptic nucleus, the medial mammillary nucleus and the lateral hypothalamus area (Fig. 7a). Double staining with anti-ChAT and anti-GABARAPL1 revealed that all cholinergic neurons within the diencephalon were GABARAPL1 positive.

#### Mesencephalon

**Figure 8:**
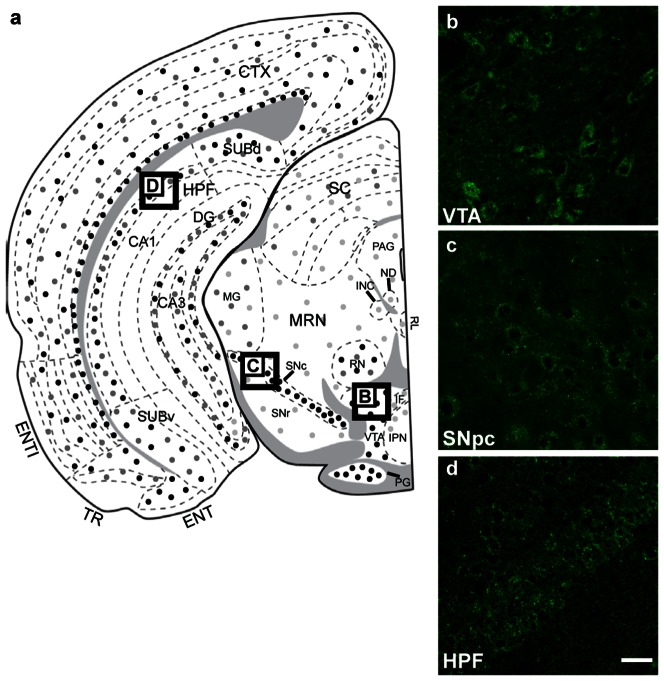
GABARAPL1 expression within the mesencephalon. Immunohistochemical analysis of WT mouse brain tissue with an anti-GABARAPL1 antibody. Displayed are representative images of the ventral tegmental area (VTA) (**b**), *the substantia nigra pars compacta* (SNpc) (**c**), and hippocampal formation (HPF) (**d**). Scale bar represents 20 µm. Abbreviations: CTX (Cortex), CA1/3 (ammon's horn, field CA1/CA3), ENT/ENTl (entorhinal area, lateral part), DG (dentate gyrus), HPF (hippocampal formation), IF (interfasicular nucleus raphé), INC (interstitial nucleus of Cajal), IPN (interpenduncular nucleus), MG (medial geniculate complex), MRN (mesenphalic reticular nucleus), ND (nucleus of Darkschewitsch), PAG (periaque ductal gray), PG (pontine gray), RL (rostral linear nucleus raphé), RN (red nucleus), SC (superior colluculus), SNc/r (*substantia nigra*, compact part/reticular part), SUBd/v (subiculum, dorsal part/ventral part), TR (post-piriform transition area), VTA (ventral tegmental area).

Weak to moderate staining was observed throughout most regions of the mesencephalon with the exception of some stronger labelling (Fig. 8a). In particular, the ventral tegmental area (VTA, Fig. 8b) and *substantia nigra* (SN, Fig. 8c) expressed a higher level of GABARAPL1. Interestingly, the GABARAPL1 expression observed in the *substantia nigra* was particularly intense in the *pars compacta* region compared to the *pars reticula* region. Double staining in these regions revealed that GABARAPL1 is found in neurons that express the dopamine transporter (DAT, Fig. 6j–l). Moderate labelling in the pyramidal layer of the hippocampal formation is depicted in Fig. 7d.

#### Rhombencephalon

**Figure 9:**
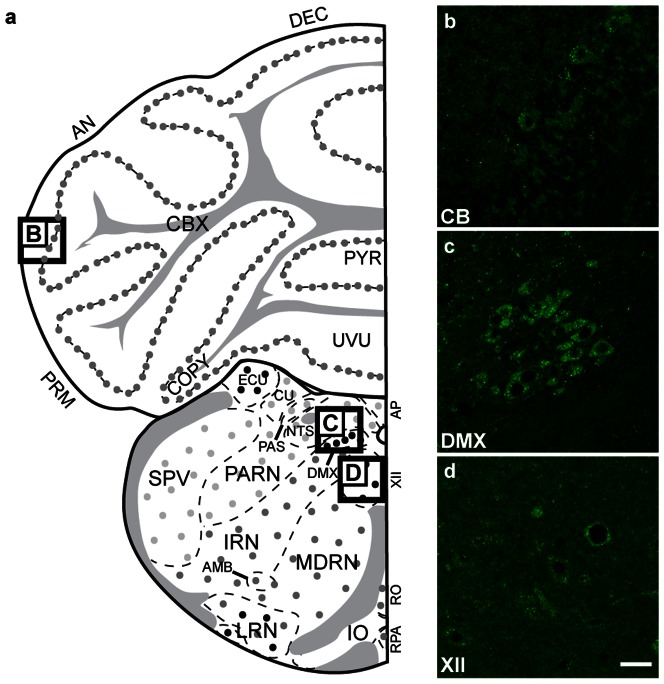
GABARAPL1 expression within the rhombencephalon. Immunohistochemical analysis of WT mouse brain tissue with an anti-GABARAPL1 antibody. Displayed are representative images of the Purkinje cells of the cerebellum (**b**), dorsal motornucleus of the vagus nerve-DMX (**c**) and hypoglossal nucleus-XII (**d**). Scale bar represents 20 µm. Abbreviations: AMB (nucleus ambiguous), AN (ansiform lobule), AP (area postrema), CBX (cerebellar cortex), COPY (copula pyramidis), CU (cuneate nucleus), DEC (declive), DMX (dorsal motornucleus of the vagus nerve), ECU (external cuneate nucleus), IO (inferior olivary complex), IRN (intermediate reticular nucleus), LRN (lateral reticular nucleus), MDRN (medullary reticular nucleus), NTS (nucleus of the solitary tract), PARN (parvicellular reticular nucleus), PAS (parasolitary nucleus), PRM (paramedian lobule), PYR (pyramus), SPV (spinal nucleus of the trigeminal), RO (nucleus raphé obscurus), RPA (nucleus raphé pallidus), UVU (uvuia), XII (hypoglossal nucleus).

GABARAPL1 labelling in the hindbrain was the strongest in the motor nuclei (Fig. 9a), in particular in the purkinje cells within the cerebellum (Fig. 9b), in the dorsal motor nucleus of the vagus nerve (DMX) (Fig. 9c) and in the hypoglossal nucleus (XII) (Fig. 9d).

### GABARAPL1 expression throughout development

**Figure 10:**
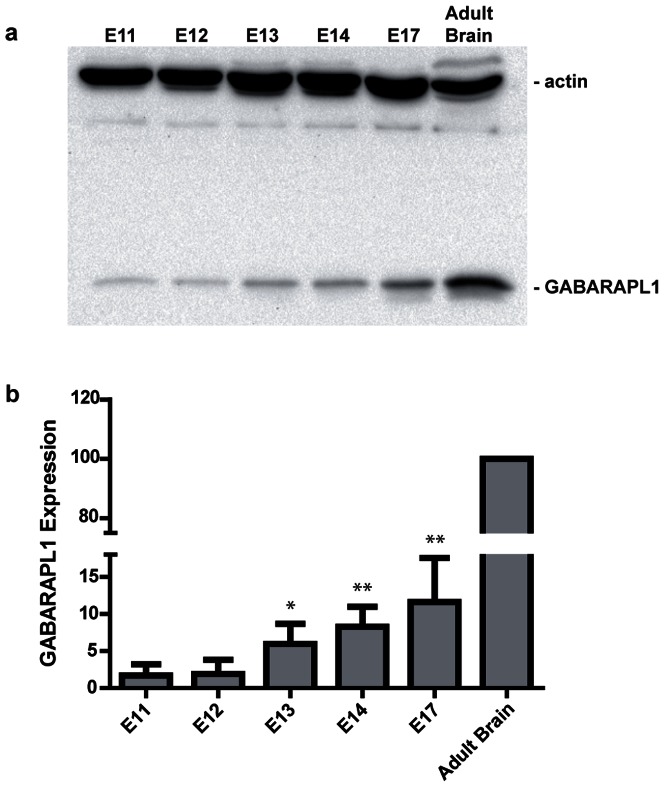
GABARAPL1 expression during embryonic development (E11 to E17). Western blotting analysis of WT mouse head tissue protein extracts using anti-GABARAPL1 and anti-Actin antibodies (**a**). Quantification of GABARAPL1 protein expression levels in the developing embryonic mouse brain (**b**). GABARAPL1 signals were normalized according to Actin amounts for each sample then displayed as a percentage of control adult brain expression. Data = mean ± SEM. **p<0.05 versus E11, *p<0.1 versus E11.

GABARAPL1 protein expression was detectable in whole head embryonic tissue by western blot analysis as early as embryonic day 11 (E11), after which it increased progressively throughout development, reaching a value six fold higher at E17 than at E11 (Fig. 10). However, expression levels of GABARAPL1 at E17, shortly before birth, were significantly inferior to GABARAPL1 expression in the adult mouse brain.

**Figure 11:**
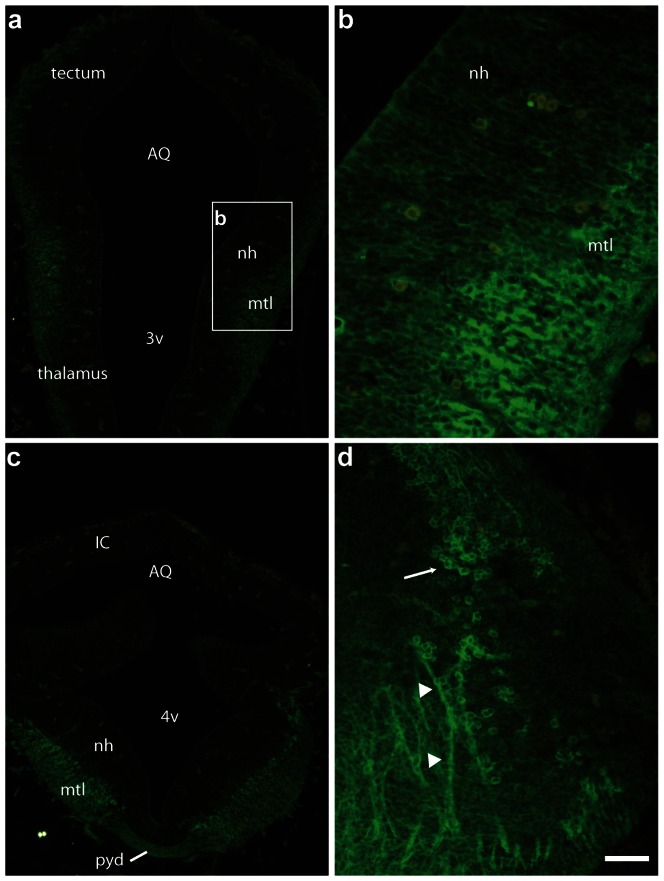
GABARAPL1 expression in the diencephalon and rhombencephalon of an E11 embryo. Videomicrographs of GABARAPL1-immunolabeled horizontal sections passing through the diencephalon (**a**, **b**) or rhombencephalon (**c**, **d**) of an E11 embryo. The GABARAPL1 immunohistochemical signal is intense in the mantle layer but is not observed in the germinal neuroepithelial layer. In the mantle layer, both immature neurons and fiber tracts are labeled. This is obvious in the mesencephalon, in which the large decussation of the pyramidal commissure is intensely labeled (**c**). At higher magnifications (**b**, **d**) the signal is detected within the cytoplasm of immature neurons (whole mantle layer in **b**, arrow in **d**), in which it is diffusely distributed, or large fibers (arrowheads in **d**). Scale bar represents 200 µm (**a**, **c**), 50 µm (**b**, **d**). Abbreviations: AQ (aqueduct), IC (inferior colliculus), mtl (mantle layer), nh (neuroepithelial layer), pyd (pyramidal tract), 3v (third ventricle), 4v (fourth ventricle).

**Figure 12:**
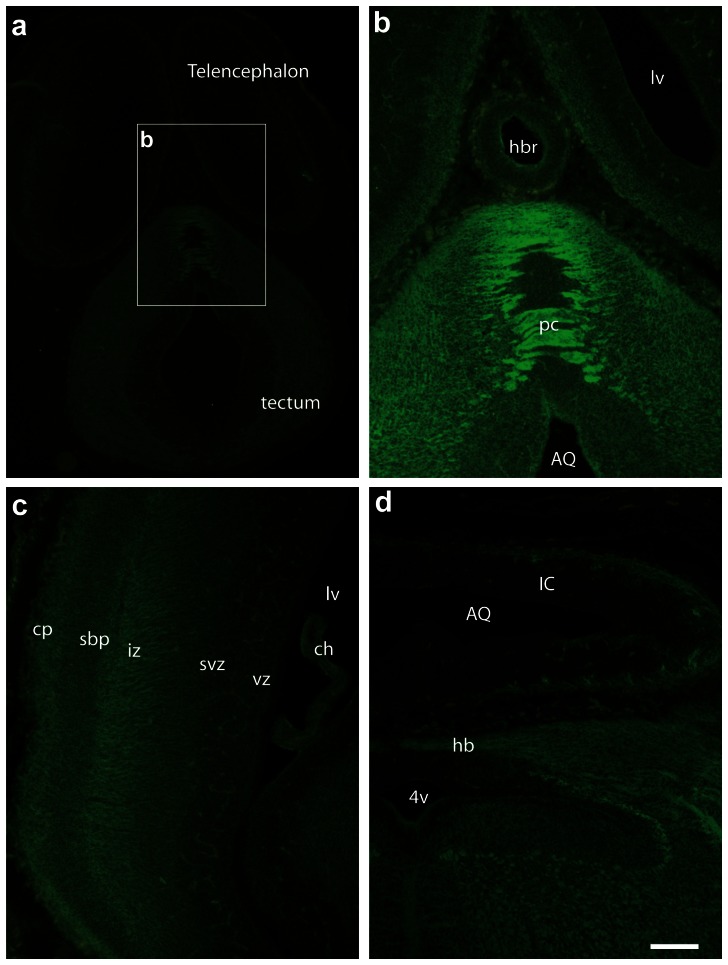
GABARAPL1 expression in the telencephalon, tectum, pallium and rhombencephalon of an E14 embryo. Videomicrographs of GABARAPL1-immunolabeled horizontal sections passing through the telencephalon and tectum (**a**, **b**), pallium (**c**) or rhombencephalon (**d**) of an E14 embryo. GABARAPL1 labeling in the mantle layer is intense in all regions, with both cell bodies and fiber tracts (posterior commissure in a or Hook bundle in **d**) displaying the immunohistochemical signal. In the pallium, ventricular and subventricular zones as well as the subplates are not intensely labeled, while the intermediate zone and cortical plates display an intense labeling. Note the absence of staining in the choroid plexus. Scale bar represents 200 µm (**a**, **c**) and 50 µm (**b**, **d**). Abbreviations: AQ (aqueduct), ch (choroid plexus), cp (cortical plate), hb (Hook bundle), hbr (habenular recess), IC (inferior colliculus), iz (intermediate zone), lv (lateral ventricle), pc (posterior commissure), sbp (subplate), svz (subventricular zone), vz (ventricular zone), 4v (fourth ventricle).

**Figure 13:**
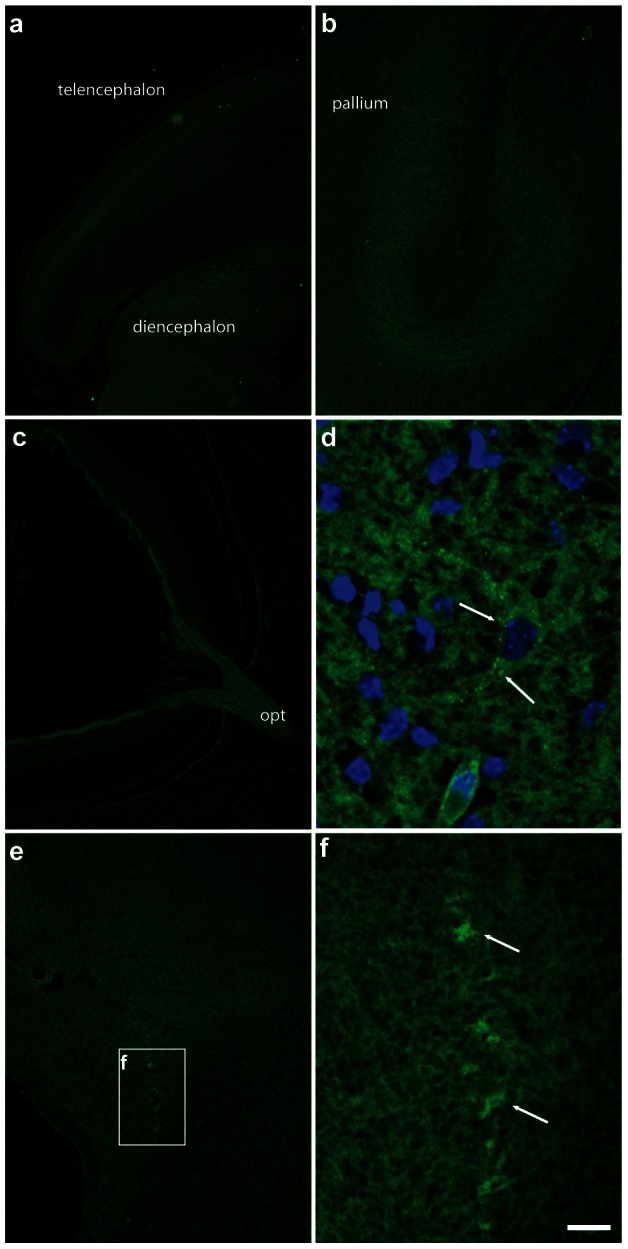
GABARAPL1 expression in the telencephalon and rhombencephalon of an E17 embryo. Videomicrographs of GABARAPL1-immunolabeled horizontal sections passing through the telencephalon (**a**, **b**), eye (**c**) or rhombencephalon (**d–f**) of an E17 embryo. GABARAPL1 labeling is present in both perikarya and fiber tracts (note the dense labeling of the optic tract as it leaves the eye. In the pallium, the intermediate zone is particularly well labeled. However, in the rhombencephalon, the labeling is less homogeneous than in earlier stages, and some cell bodies display a labeling which is more intense than in surrounding tissue, indicating that the adult pattern is beginning to differentiate. This is clear in the motoneurons of the mesencephalic nucleus of the trigeminal nerve (e, arrows in **f**). A punctate-like staining also became visible at this stage (arrows in **d**). Scale bar represents 300 µm (**a**), 100 µm (**b**, **c**, **e**), 20 µm (**f**) ) and 10 µm (**d**). Abbreviations: iz (intermediate zone), opt (optic tract).

In accordance with western blot results, GABARAPL1 staining was present at E11 to E17 throughout the developing brain from the telencephalon to the brainstem (Fig. 11, 12, 13). GABARAPL1 labelling became ubiquitous throughout the brain as the mantle layer differentiated. This labelling was visible throughout the mantle layer in the pallium but never in the neuroepithelial layer of the developing brain (Fig. 11a, b 12c and 13a–b). This result is in accordance with the presence of GABARAPL1 in immature neurons.

During earlier developmental stages (E11/E12) cells in the mantle layer were uniformly labelled for GABARAPL1 (Fig. 11a, b). Differential patterns of GABARAPL1 labelling only started to become visible at intermediate stages (E14, Fig. 12) and were more obvious at later stages (E17, Fig. 13), at which point the adult expression pattern appeared to develop. This differential pattern similar to the adult was particularly evident in the motoneurons, such as surrounding the 4^th^ ventricle in the brainstem (Fig. 12d, Fig. 13e, f). In the pallium, organized layers of GABARAPL1 labelling were visible, in which the intermediate zone and cortical plate strongly expressed the GABARAPL1 protein (Fig. 12c or 13a, b).

Contrary to the adult mouse, we also observed a labelling of fibers in the embryo as is visible in the pyramidal tract at E11 (Fig. 11c), the posterior commissure at E14 (Fig. 12a, b) and the optic tract at E17 (Fig. 13 c). Fiber labelling with the anti-GABARAPL1 was also demonstrated in the intermediate zone of the cortical plate, which gives rise to the corpus callosum (Fig. 12c, 13a, b).

At a cellular level, GABARAPL1 labelling appeared diffused throughout the cell at earlier stages of development and, as mentioned before, only showed signs of the stronger punctate-like structures visible in the cytoplasm at later stages, around E17 (Fig. 13d).

## Discussion

Our study is the first to map out the differential expression of the GABARAPL1 protein in the adult and developing embryonic murine brain. In this study, we confirmed the strong presence of this protein throughout the brain, visible in immature neurons and fibers during differentiation and in mature neurons in the adult.

Immunohistochemical labelling with a specific anti-GABARAPL1 antibody revealed an overall similar distribution in the adult of the mouse GABARAPL1 protein compared to that of the rat *gabarapl1* mRNA [14], suggesting that in this case, mRNA levels do depict protein levels. Examples of these coherences include the cerebral cortex, which was strongly labelled in both studies, and the pallidum, which was weakly labelled in both studies. The similar distribution of *gabarapl1* mRNA and protein is particularly evident through the labelling pattern of the *substantia nigra*, in which the *pars compacta* was particularly strongly labelled with both the *gabarapl1* mRNA probe [14] and the GABARAPL1 protein specific antibody, while the *pars reticula* remained less strongly labelled in both cases.

Despite the overall similar distribution between the GABARAPL1 protein and its mRNA, some variations were observed. These differences are due either to a difference between mRNA and protein expression levels or between species. For example, in the telencephalon, the relative protein expression of GABARAPL1 was found to be lower than its relative mRNA levels in the main olfactory bulb, the anterior olfactory nucleus, the fundus of the striatum and the bed nucleus of the accessory olfactory tract.

A few dissimilarities were visible between the two species: rat and mouse. *gabarapl1* mRNA expression in the induseum griseum of the cortex and certain regions of the diencephalon varied. In the induseum griseum, intense mRNA staining was observed in the rat and weak mRNA staining, associated with weak to moderate protein expression, were observed in the mouse. The mRNA expression and corresponding protein expression of GABARAPL1 in the mouse was found to be weak in several sensory nuclei of the thalamus as well as in the *zona incerta*, and several hypothalamic nuclei compared to a higher expression observed in the rat. On the other hand, *gabarapl1* expression appeared to be higher in the mouse anteroventral periventricular hypothalamic nucleus compared its expression in the rat. Lastly, we also observed a lower expression of *gabarapl1* in the mouse interstitial nucleus of Cajal and the nucleus of Darkschewitsch and a higher expression in the external cuneate nucleus compared to the mRNA expression in the rat.

What was particularly interesting in the adult was the strong expression of GABARAPL1 restricted to neuronal cells. Since GABARAPL1 is an important effector of the autophagic process, and autophagy is a globally important cellular process, one would expect to still see some labelling in the surrounding cells. This difference could be explained by the higher importance of housekeeping autophagy in post-mitotic cells, such as neurons, compared to their surrounding supporting cells, such as glia. Not only did GABARAPL1 display a differential expression between different cells types, but its expression also varied in different brain regions. By use of double labelling with different antibodies, we demonstrated that GABARAPL1 is expressed in different types of neurons, including both GABAergic (calbindin and parvalbumin positive neurons in the pallidum) and glutamatergic (pyramidal cells of the cortex) neurons. Double labelling experiments with an anti-DAT also revealed that GABARAPL1 punctate staining was strong in dopaminergic neurons throughout the *substantia nigra* as well as the ventral tegmental area. While GABARAPL1 was present in different neuronal types, the level of its expression varied from one region to another. For example, GABARAPL1 labeling was visible in the GABAergic Purkinje cells but absent from the glutamatergic granule cells of the cerebellum. GABARAPL1 labeling was also visible in the pyramidal and granular layers (containing excitatory neurons) of the hippocampus and dentate gyrus but not in the molecular layers (composed of inhibitory interneurons). More detailed studies of GABARAPL1 expression with respect to the organization of specific brain regions combined with functional approaches will therefore be interesting for future studies.

To date, no other mammalian Atg8 protein homologue has been mapped out in the adult brain, although their mRNA expression levels have been observed [13], [14]. As such, protein mapping of the other members of the GABARAP and LC3 families in the brain would be very interesting in order to determine if there is a compensation of the other members in the cells in which GABARAPL1 is less strongly expressed. This map of basal levels of GABARAPL1 in the brain is also a valuable tool that can be used to compare GABARAPL1 protein levels under different conditions such as neurodegenerative models using different chemical molecules, experimentation methods or knockout models, such as the neuronal specific *atg5* and *atg7* mouse knockout models [28], [29].

In the embryo, similar GABARAPL1 distribution patterns compared to the adult became visible at E14 and even more pronounced at E17, at which point neuroblast expression of GABARAPL1 corresponded to the adult expression of this protein. Unlike in the adult, however, GABARAPL1 staining was also observed in both fibers. In particular, a strong signal was detected in fiber tracts at E11, suggesting a role for GABARAPL1 in axonal growth and elongation during development. Indeed, GABARAPL1, like GABARAP [30] and LC3 [31], is a microtubule-associated protein known to bind to tubulin and promote tubulin assembly and microtubule bundling [22] and, therefore, most likely plays an important role in axon and dendritic projection during development. Contrary to the mantle layer, no labelling in the neuroepithellium was visible, suggesting that GABARAPL1 does not play a role in stem cells surrounding the ventricles.

*lc3α* and *lc3ß* mRNA expression have also been examined in the developing murine central nervous system [8]. In these studies, the authors demonstrate identification of *lc3 in-situ* staining in the forebrain, midbrain and commissural neurons of the developing dorsal neural tube, similar to the expression we observed for the GABARAPL1 protein.

In the adult and later stages of development, GABARAPL1 labelling displays a weak signal in the cytoplasm and some neuronal extensions with brighter punctate structures throughout the cytoplasm and a complete absence of labelling in the nucleus. This staining is consistent with the GABARAPL1 distribution we have previously observed in our *in vitro* models when studying the role of GABARAPL1 in autophagy [20]. The importance of a basal amount of autophagy in neurons has been demonstrated by the two conditional knockouts: *atg5*−/− and *atg7*−/− [28], [29]. In both these mouse models, a deficiency in autophagy in the central nervous system resulted in severe neurodegeneration. As such, one would expect to see a basal level of autophagy visible in the murine brain, suggesting that the punctate structures visible with our GABARAPL1 staining likely, at least partially, represent autophagosomal membranes. Indeed, primary cortical cultures (E17) incubated with or without Baf A1, an inhibitor of the autophagic flux described to inhibit the fusion of autophagosomes with lysosomes, revealed a significant increase in the number of intracellular vesicles positive for GABARAPL1 and/or p62, a described substrate of autophagy. These *ex vivo* data demonstrate that GABARAPL1 is, at least partially, associated with autophagosomes in primary neurons and consequently in the mouse brain.

Nonetheless, some vesicles are positive for both proteins while others are only positive for GABARAPL1 or p62. The p62 only positive vesicles are likely to be autophagosomes containing none or little GABARAPL1. Indeed, we are aware of the existence of 6 members of the Atg8 family, most of which are expressed in neurons to different extents, and it is still unknown if they all participate in the formation of each autophagosome or, more likely, if they are specific to different structures.

Interestingly, while LC3 expression has been observed in the murine brain, the GFP-LC3 mouse does not display any kind of punctate structure in the brain [25], [29], [31]. This differs from GABARAPL1, which does display a strong punctate labelling, and may suggest that GABARAPL1 plays a more prominent role in the autophagic process under normal conditions in the brain. This question will only be answered with the creation of a knockout mouse model for GABARAPL1. Indeed, the study of two previous knockout models against LC3B and GABARAP did not portray autophagic deregulation or pathologies linked to the disruption of the autophagosome/lysosome pathway [8], [9], suggesting that a compensatory mechanism by the other members of the family is in place. Since GABARAPL1 is predominant in the mouse brain and it interacts with several adaptor proteins for selective autophagy, which degrades unwanted protein aggregates and damaged organelles, such as p62, NBR1 (neighbor of BRCA1) and NIX (NIP3-like protein X ) [32], [33], [34], its extinction might present a more severe phenotype, especially in this organ.

GABARAPL1 has also been shown to bind to and is potentially implicated in trafficking of the GABA_A_R (gamma-aminobutyric acid type A receptor) [22]. Regulation of GABA_A_R numbers and clustering is important for modulation of synaptic strength at inhibitory synapses, which is mediated by an accumulation of the GABA_A_R at the postsynaptic dendrite to maintain neural network homeostasis [35]. Given that GABARAPL1 interacts with this receptor and is expressed in GABAergic neurons, it may also play a role for in neural network homeostasis. GABARAPL1 expression in GABAergic neurons becomes particularly interesting for studies of depression. Indeed, *gabarapl1* and *GABA_A_R* were found to be upregulated in the hippocampus and certain regions of the cortex of human brains affected by major depression regulated [36]. kappa opioid receptor (KOR)-induced signalling has also been implicated in depression-like behaviour [37]. Since *gabarapl1* is upregulated in major depression and GABARAPL1 interacts with and mediates KOR expression [21], then another of its functions might be to increase the transport of KOR to the plasma membrane to enhance depressive-like behaviour.

Conversely, a suppression of GABAergic inhibition has been linked to the etiology of some neurodevelopmental disorders, such as autism [38] and schizophrenia [39]. In fact, *gabarapl1* was found to be deleted in a genome wide experiment using in an European ancestry case-control data set for autism [40].

*gabarapl1* expression is also shown to be deregulated in Parkinson's disease. *gabarapl1* is downregulated in a MPTP model of Parkinson's in macaque monkeys [16] as well as in the dopaminergic neurons of the *SNpc* of Parkinson's patients [17], a region of the brain in which we have found a high expression of the GABARAPL1 protein. Moreover, GABARAPL1 can interact with α-synuclein, a protein shown to form protein aggregates and deleterious lewy bodies in Parkinson's disease, and displays a higher binding affinity for α-synuclein oligomers compared to monomers [41]. GABARAPL1 may, therefore, play a protective role in this disease by aiding in the degradation of unwanted α-synuclein aggregates until such time as it is downregulated with the progression of the disease. Indeed, *gabarapl1* transcription is upregulated in the presence of estrogens [42] and estrogen has been show to have a protective effect on an MPTP murine model of Parkinson's disease [43].

Overall, our study describes, for the first time, the protein expression map of an autophagy factor in the adult and embryonic murine brain. Its expression and cellular localization is consistent with previous data obtained relating to its mRNA expression in the rat, suggesting a similar expression and role in different mammals. Its expression is also consistent with its involvement in receptor transport and autophagy. Moreover, its high expression in the brain and in particular in disease-linked areas such as the *substantia nigra pars compacta* might set up GABARAPL1 as a potential therapeutic target for diverse neurological disorders.

## References

[1] Klionsky DJ, Emr SD (2000) Autophagy as a regulated pathway of cellular degradation. Science 290: 1717–1721. 11099404 10.1126/science.290.5497.1717 PMC2732363

[2] Levine B, Klionsky DJ (2004) Development by self-digestion: molecular mechanisms and biological functions of autophagy. Dev Cell 6: 463–477. 15068787 10.1016/s1534-5807(04)00099-1

[3] Cuervo AM (2004) Autophagy: many paths to the same end. Mol Cell Biochem 263: 55–72. 10.1023/B:MCBI.0000041848.57020.57 27520665

[4] Shintani T, Klionsky DJ (2004) Autophagy in health and disease: a double-edged sword. Science 306: 990–995. 15528435 10.1126/science.1099993 PMC1705980

[5] Ichimura Y, Imamura Y, Emoto K, Umeda M, Noda T, et al (2004) In vivo and in vitro reconstitution of Atg8 conjugation essential for autophagy. J Biol Chem 279: 40584–40592. 15277523 10.1074/jbc.M405860200

[6] Nakatogawa H, Ichimura Y, Ohsumi Y (2007) Atg8, a ubiquitin-like protein required for autophagosome formation, mediates membrane tethering and hemifusion. Cell 130: 165–178. 17632063 10.1016/j.cell.2007.05.021

[7] Weidberg H, Shvets E, Shpilka T, Shimron F, Shinder V, et al (2010) LC3 and GATE-16/GABARAP subfamilies are both essential yet act differently in autophagosome biogenesis. EMBO J 29: 1792–1802. 20418806 10.1038/emboj.2010.74 PMC2885923

[8] Cann GM, Guignabert C, Ying L, Deshpande N, Bekker JM, et al (2008) Developmental expression of LC3alpha and beta: absence of fibronectin or autophagy phenotype in LC3beta knockout mice. Dev Dyn 237: 187–195. 18069693 10.1002/dvdy.21392

[9] O'Sullivan GA, Kneussel M, Elazar Z, Betz H (2005) GABARAP is not essential for GABA receptor targeting to the synapse. Eur J Neurosci 22: 2644–2648. 16307606 10.1111/j.1460-9568.2005.04448.x

[10] Pellerin I, Vuillermoz C, Jouvenot M, Ordener C, Royez M, et al (1993) Identification and characterization of an early estrogen-regulated RNA in cultured guinea-pig endometrial cells. Mol Cell Endocrinol 90: R17–21. 8495796 10.1016/0303-7207(93)90161-c

[11] Sagiv Y, Legesse-Miller A, Porat A, Elazar Z (2000) GATE-16, a membrane transport modulator, interacts with NSF and the Golgi v-SNARE GOS-28. EMBO J 19: 1494–1504. 10747018 10.1093/emboj/19.7.1494 PMC310219

[12] Nemos C, Mansuy V, Vernier-Magnin S, Fraichard A, Jouvenot M, et al (2003) Expression of gec1/GABARAPL1 versus GABARAP mRNAs in human: predominance of gec1/GABARAPL1 in the central nervous system. Brain Res Mol Brain Res 119: 216–219. 14625090 10.1016/j.molbrainres.2003.09.011

[13] Mansuy-Schlick V, Tolle F, Delage-Mourroux R, Fraichard A, Risold PY, et al (2006) Specific distribution of gabarap, gec1/gabarap Like 1, gate16/gabarap Like 2, lc3 messenger RNAs in rat brain areas by quantitative real-time PCR. Brain Res 1073–1074: 83–87. 10.1016/j.brainres.2005.11.004 16458273

[14] Tolle F, Risold PY, Mansuy-Schlick V, Rossi E, Boyer-Guittaut M, et al (2008) Specific regional distribution of gec1 mRNAs in adult rat central nervous system. Brain Res 1210: 103–115. 18423580 10.1016/j.brainres.2008.02.077

[15] Wang Y, Dun SL, Huang P, Chen C, Chen Y, et al (2006) Distribution and ultrastructural localization of GEC1 in the rat CNS. Neuroscience 140: 1265–1276. 16650615 10.1016/j.neuroscience.2006.03.013

[16] Storvik M, Arguel MJ, Schmieder S, Delerue-Audegond A, Li Q, et al (2010) Genes regulated in MPTP-treated macaques and human Parkinson's disease suggest a common signature in prefrontal cortex. Neurobiol Dis 38: 386–394. 20206263 10.1016/j.nbd.2010.02.008

[17] Simunovic F, Yi M, Wang Y, Macey L, Brown LT, et al (2009) Gene expression profiling of substantia nigra dopamine neurons: further insights into Parkinson's disease pathology. Brain : a journal of neurology 132: 1795–1809. 19052140 10.1093/brain/awn323 PMC2724914

[18] Le Grand JN, Chakrama FZ, Seguin-Py S, Fraichard A, Delage-Mourroux R, et al (2011) GABARAPL1 antibodies: Target one protein, get one free!. Autophagy 7: 1302–1307. 21862879 10.4161/auto.7.11.16723

[19] Brischoux F, Fellmann D, Risold PY (2001) Ontogenetic development of the diencephalic MCH neurons: a hypothalamic ‘MCH area’ hypothesis. Eur J Neurosci 13: 1733–1744. 11359525 10.1046/j.0953-816x.2001.01552.x

[20] Chakrama FZ, Seguin-Py S, Le Grand JN, Fraichard A, Delage-Mourroux R, et al (2010) GABARAPL1 (GEC1) associates with autophagic vesicles. Autophagy 6: 495–505. 20404487 10.4161/auto.6.4.11819

[21] Chen C, Li JG, Chen Y, Huang P, Wang Y, et al (2006) GEC1 interacts with the kappa opioid receptor and enhances expression of the receptor. J Biol Chem 281: 7983–7993. 16431922 10.1074/jbc.M509805200

[22] Mansuy V, Boireau W, Fraichard A, Schlick JL, Jouvenot M, et al (2004) GEC1, a protein related to GABARAP, interacts with tubulin and GABA(A) receptor. Biochem Biophys Res Commun 325: 639–648. 15530441 10.1016/j.bbrc.2004.10.072

[23] Friedman LG, Lachenmayer ML, Wang J, He L, Poulose SM, et al (2012) Disrupted autophagy leads to dopaminergic axon and dendrite degeneration and promotes presynaptic accumulation of alpha-synuclein and LRRK2 in the brain. J Neurosci 32: 7585–7593. 22649237 10.1523/JNEUROSCI.5809-11.2012 PMC3382107

[24] Peng C, Ye J, Yan S, Kong S, Shen Y, et al (2012) Ablation of vacuole protein sorting 18 (Vps18) gene leads to neurodegeneration and impaired neuronal migration by disrupting multiple vesicle transport pathways to lysosomes. J Biol Chem 287: 32861–32873. 22854957 10.1074/jbc.M112.384305 PMC3463306

[25] Mizushima N, Yamamoto A, Matsui M, Yoshimori T, Ohsumi Y (2004) In vivo analysis of autophagy in response to nutrient starvation using transgenic mice expressing a fluorescent autophagosome marker. Mol Biol Cell 15: 1101–1111. 14699058 10.1091/mbc.E03-09-0704 PMC363084

[26] Yamamoto A, Tagawa Y, Yoshimori T, Moriyama Y, Masaki R, et al (1998) Bafilomycin A1 prevents maturation of autophagic vacuoles by inhibiting fusion between autophagosomes and lysosomes in rat hepatoma cell line, H-4-II-E cells. Cell Struct Funct 23: 33–42. 9639028 10.1247/csf.23.33

[27] Korolchuk VI, Mansilla A, Menzies FM, Rubinsztein DC (2009) Autophagy inhibition compromises degradation of ubiquitin-proteasome pathway substrates. Mol Cell 33: 517–527. 19250912 10.1016/j.molcel.2009.01.021 PMC2669153

[28] Hara T, Nakamura K, Matsui M, Yamamoto A, Nakahara Y, et al (2006) Suppression of basal autophagy in neural cells causes neurodegenerative disease in mice. Nature 441: 885–889. 16625204 10.1038/nature04724

[29] Komatsu M, Waguri S, Chiba T, Murata S, Iwata J, et al (2006) Loss of autophagy in the central nervous system causes neurodegeneration in mice. Nature 441: 880–884. 16625205 10.1038/nature04723

[30] Wang H, Olsen RW (2000) Binding of the GABA(A) receptor-associated protein (GABARAP) to microtubules and microfilaments suggests involvement of the cytoskeleton in GABARAPGABA(A) receptor interaction. J Neurochem 75: 644–655. 10899939 10.1046/j.1471-4159.2000.0750644.x

[31] Mann SS, Hammarback JA (1994) Molecular characterization of light chain 3. A microtubule binding subunit of MAP1A and MAP1B. J Biol Chem 269: 11492–11497. 7908909

[32] Pankiv S, Clausen TH, Lamark T, Brech A, Bruun JA, et al (2007) p62/SQSTM1 binds directly to Atg8/LC3 to facilitate degradation of ubiquitinated protein aggregates by autophagy. J Biol Chem 282: 24131–24145. 17580304 10.1074/jbc.M702824200

[33] Larsen KB, Lamark T, Overvatn A, Harneshaug I, Johansen T, et al (2010) A reporter cell system to monitor autophagy based on p62/SQSTM1. Autophagy 6: 784–793. 20574168 10.4161/auto.6.6.12510

[34] Rual JF, Venkatesan K, Hao T, Hirozane-Kishikawa T, Dricot A, et al (2005) Towards a proteome-scale map of the human protein-protein interaction network. Nature 437: 1173–1178. 16189514 10.1038/nature04209

[35] Rannals MD, Kapur J (2011) Homeostatic strengthening of inhibitory synapses is mediated by the accumulation of GABA(A) receptors. J Neurosci 31: 17701–17712. 22131430 10.1523/JNEUROSCI.4476-11.2011 PMC3396123

[36] Sequeira A, Mamdani F, Ernst C, Vawter MP, Bunney WE, et al (2009) Global brain gene expression analysis links glutamatergic and GABAergic alterations to suicide and major depression. PLoS One 4: e6585. 19668376 10.1371/journal.pone.0006585 PMC2719799

[37] Knoll AT, Carlezon WA (2010) Dynorphin, stress, and depression. Brain Res 1314C: 56. 10.1016/j.brainres.2009.09.074 PMC2819644 19782055

[38] Hussman JP (2001) Suppressed GABAergic inhibition as a common factor in suspected etiologies of autism. J Autism Dev Disord 31: 247–248. 11450824 10.1023/a:1010715619091

[39] Lewis DA, Hashimoto T, Volk DW (2005) Cortical inhibitory neurons and schizophrenia. Nat Rev Neurosci 6: 312–324. 15803162 10.1038/nrn1648

[40] Griswold AJ, Ma D, Cukier HN, Nations LD, Schmidt MA, et al (2012) Evaluation of copy number variations reveals novel candidate genes in autism spectrum disorder-associated pathways. Hum Mol Genet 10.1093/hmg/dds164 PMC3392110 22543975

[41] Schnack C, Danzer KM, Hengerer B, Gillardon F (2008) Protein array analysis of oligomerization-induced changes in alpha-synuclein protein-protein interactions points to an interference with Cdc42 effector proteins. Neuroscience 154: 1450–1457. 18541383 10.1016/j.neuroscience.2008.02.049

[42] Vernier-Magnin S, Muller S, Sallot M, Radom J, Musard JF, et al (2001) A novel early estrogen-regulated gene gec1 encodes a protein related to GABARAP. Biochem Biophys Res Commun 284: 118–125. 11374880 10.1006/bbrc.2001.4908

[43] Bourque M, Dluzen DE, Di Paolo T (2009) Neuroprotective actions of sex steroids in Parkinson's disease. Frontiers in neuroendocrinology 30: 142–157. 19410597 10.1016/j.yfrne.2009.04.014

